# A Rare Presentation of Lyme Disease in an Immunocompromised Patient

**DOI:** 10.7759/cureus.58605

**Published:** 2024-04-19

**Authors:** Eric J Basile, Madeline Smoot, Megan E Hanna, Zohaib Ijaz, Ellen C Keeley

**Affiliations:** 1 Internal Medicine, University of Florida College of Medicine, Gainesville, USA; 2 Cardiovascular Medicine, University of Florida Health, Gainesville, USA

**Keywords:** lyme disease, lyme, heart failure with reduced ejection fraction, heart failure, lyme carditis

## Abstract

Lyme disease is a progressive infectious disease caused by the *Borrelia* species that affects multiple organ systems, including the brain, heart, skin, and musculoskeletal systems. The cardiac manifestations of Lyme disease typically present with atrioventricular nodal conduction abnormalities and, more rarely, myocarditis. We report a case of an immunocompromised 57-year-old woman who presented with acute onset shortness of breath, hypervolemia, injective conjunctiva, and global vision loss of the left eye in the setting of a recent tick bite. Serologic testing confirmed borreliosis, and cardiac testing demonstrated acute isolated systolic heart failure without any cardiac conduction system abnormalities on the electrocardiogram. The diagnosis of Lyme carditis was made, and the patient was started on doxycycline with complete recovery of cardiac systolic function. This case demonstrates atypical cardiac manifestations of Lyme disease and highlights the difficulty in workup and understanding of Lyme carditis particularly in immunocompromised patients.

## Introduction

Lyme carditis is seen in 1.5%-10% of all patients with Lyme borreliosis in the United States, with the majority developing cardiac conduction abnormalities and 60% developing signs of myopericarditis [1-3]. Cardiac symptoms typically begin approximately 21 days after the appearance of erythema chronicum migrans. In exceedingly rare cases, cardiac manifestations of Lyme disease have included myocarditis, pericarditis, pancarditis, and dilated cardiomyopathy [4]. There is even evidence to suggest that these conditions may be the only presenting signs in a patient, particularly those who are immunocompromised [5].

Workup for Lyme carditis includes a 12-lead electrocardiogram (ECG) with or without a 24-hour Holter ECG for the detection of PR prolongation and chest radiography and echocardiography for the detection of regional/global wall motion abnormalities. Serologic workup includes two-tiered testing of enzyme-linked immunosorbent assay (ELISA), followed by a Western immunoblot to confirm the infection. ELISA testing may be negative in the early phases of Lyme disease in immunocompromised patients [2]. An IgM Western immunoblot is considered positive if two or more bands are present. There is a significant false positive rate due to cross-reactivity with autoimmune disorders and other infectious organisms [4]. Due to the variability of serologic and immunoassays, all test results should be correlated with the patient’s history and clinical presentation. The goal of treatment for Lyme is the elimination of the infectious organism, usually with antimicrobials such as doxycycline [6].

In the presented case, we outline a rare case of myocarditis-predominant Lyme carditis without cardiac conduction abnormalities in an immunodeficient host. This case highlights the ambiguity in testing and presentation while underscoring the importance of recognizing uncommon Lyme-associated cardiac sequelae.

## Case presentation

The patient is a 57-year-old woman who was admitted to the cardiology teaching service at the University of Florida in Gainesville, Florida, for acute heart failure with reduced ejection fraction. The patient's past medical history was significant for human immunodeficiency virus (HIV) with a recent CD4 count of 253 cells/mm^3^, tuberculosis treated for nine months, prior injection drug use, bipolar disorder, chronic obstructive pulmonary disease, type 2 diabetes mellitus, hypertension, and atrial fibrillation/flutter with previous cardioversion. Initial presenting symptoms included subacute arthralgias, shortness of breath, bilateral lower extremity swelling, exertional chest pain, dyspnea on exertion, a productive cough, subjective fevers, and unilateral vision loss. Upon history, the patient revealed that she was a lifelong resident of Florida with minimal travel exposure and has not been compliant with her home Biktarvy for more than one month; additionally, she had removed an engorged tick approximately one month before her admission at which point she had an erythematous rash at the site of the tick bite. She has not had any alcohol intake in a year, nor has she used tobacco or illicit substances. The signs and symptoms subsequently developed within one to three weeks after removal of the tick.

**Figure 1:**
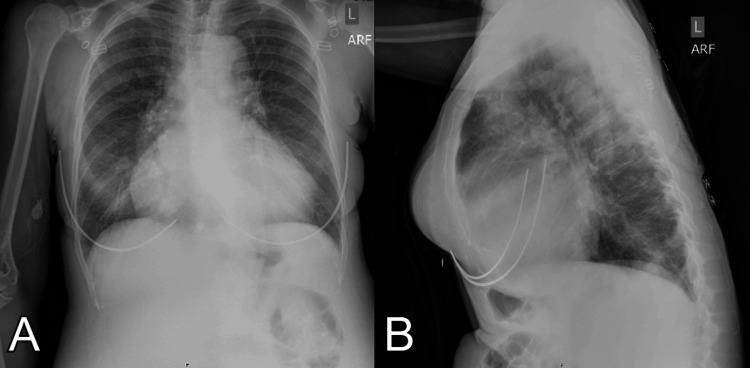
Anterior-posterior (A) and lateral (B) chest X-rays demonstrating pulmonary vascular congestion, cephalization of vasculature, and bilateral infiltrates most consistent with pulmonary edema.

On physical examination, the patient had non-purulent injected conjunctiva on the left side with associated global vision loss. Chest X-ray showed an enlarged cardiac silhouette with cardiogenic pulmonary edema, and ECG revealed diffuse ST-segment depressions (Figure 1). Her initial BNP level was greater than 2,400 pg/mL, and troponins were moderately elevated to 260 ng/L. CRP was 12.85. Her most recent CD4 level was 253 cells/mm^3^, and her HIV viral load was 360,000 copies/mL. Ophthalmology and infectious disease services were consulted to provide recommendations on evaluation and management.

Ophthalmology noted subconjunctival hemorrhage, diffuse corneal edema, endothelial deposits in Arlt's triangle, 2+ cells and flare without hypopyon in the anterior chamber, and posterior synechiae. Ophthalmology performed an ultrasound of the eye with vitreous cultures and intraocular injection of vancomycin and ceftazidime empirically. The vitreous cultures, including Gram stain and fungal culture, were negative. The ophthalmology service recommended obtaining assays for *Toxoplasma*, QuantiFERON gold release assay, an autoimmune panel, herpes simplex virus 1 and 2 (HSV1 and HSV2), varicella-zoster virus (VZV), Epstein-Barr virus (EBV), cytomegalovirus (CMV), *Chlamydia*,* Neisseria*,and syphilis - all of which were negative. They then recommended starting atropine three times per day, prednisone eyedrops every hour, and oral prednisone 50 mg with a taper. Infectious disease recommended re-initiation of the patient's home Biktarvy and workup for tickborne illness. Serologies were sent, and the IgM Lyme was positive. The IgM Western immunoblot was positive for 23 kDa, 39 kDa, and 41 kDa bands. The IgG Lyme was negative, as was the ELISA.

**Figure 2:**
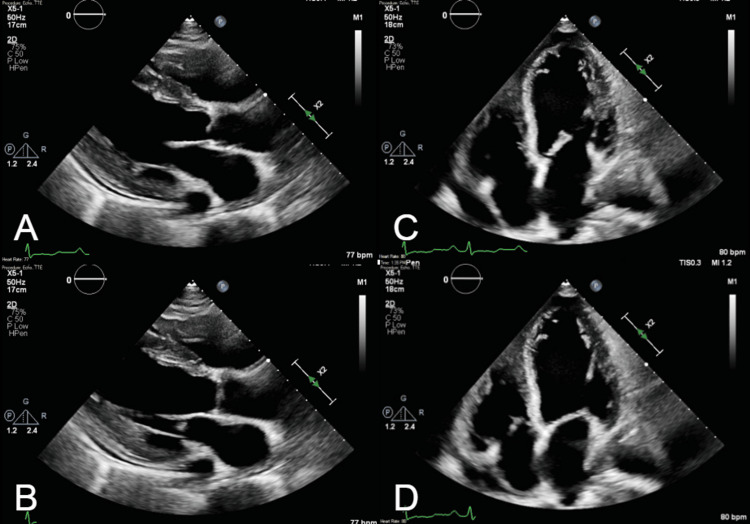
Parasternal long axis (A, B) and apical four-chamber (C, D) transthoracic echocardiography demonstrating a reduced ejection fraction of 20% with severe biatrial and biventricular dilatation.

The patient received intravenous diuretics for the management of her hypervolemia. A transthoracic echocardiogram revealed an estimated left ventricular ejection fraction (LVEF) of 20% with severe biatrial and biventricular dilatation (Figure 2). As the reduction in LVEF was new, an ischemic workup was pursued; the patient underwent a nuclear stress test, which was negative for inducible ischemia. Furthermore, the patient had not been consuming alcohol or illicit substances that could contribute to cardiomyopathy. Given the constellation of symptoms - panuveitis, joint pain, unspecified rashes, new heart failure, and positive Lyme serologies - the diagnosis of Lyme carditis was made. She underwent treatment with guideline-directed medical therapy, in addition to doxycycline 100 mg twice per day for 21 days and prednisone 50 mg with a taper. She received the first four doses of each in the hospital. The patient experienced hot flashes with increased erythema and pruritus of her lower extremities. Her vision began to improve after 48 hours, as well as her dyspnea on exertion and joint pain. She was treated with aspirin and diphenhydramine. The patient was later discharged with plans to follow up with cardiology, infectious disease, and ophthalmology in the outpatient setting. A repeat TTE performed at an outside clinic demonstrated a recovery in LVEF back to normal 55% with decreased dilation of all cardiac chambers. All previous signs and symptoms resolved upon completion of the antibiotic regimen and steroid taper. She did not show up to the ophthalmology appointment and was ultimately lost to follow-up.

## Discussion

One rare and reversible etiology of acute-onset congestive heart failure is *Borrelia burgdorferi* infection. There are only a few reported cases of Lyme carditis-associated heart failure without conduction abnormalities. Because approximately 90% of Lyme carditis cases present with conduction abnormalities, it is often not considered in the differential for patients without AV node conduction abnormalities [2,3].

In the case presented here, a woman presented with multiple nonspecific findings including left eye panuveitis with associated vision loss, arthralgias, bilateral popliteal tenderness, nonspecific erythematous lesions, and shortness of breath in the setting of uncontrolled HIV. The patient initially tested negative on ELISA for Lyme; as ELISA is more likely to be negative in early disease and in immunosuppressed/compromised patients, IgM specific for *B. burgdorferi* was sent and came back positive. Importantly, the Western blot is also more specific than ELISA or an immunofluorescent assay, though there is a significant false positive rate due to cross-reactivity with autoimmune disorders and other infectious organisms such as EBV, CMV, bacterial endocarditis, syphilis, *Helicobacter pylori*, and other spirochetal organisms [2]. As such, further testing was conducted and demonstrated positive IgM for HSV 1/2 and HIV 1/2. A Western blot was performed, which demonstrated positivity among three *Borrelia*-specific bands (41 kDa, 23 kDa, and 39 kDa). The 41 kDa band correlates with a bacterial flagellum and has a high false-positive rate as even colonization can produce positive results. The 23 kDa band is more specific for *B. burgdorferi* and has a lower false-positive rate than that of the 41 kDa band. The 39 kDa band has the highest correlation with early-stage Lyme disease and is therefore the most specific of the panel for *B. burgdorferi *infection [2]. Coincidentally, IgM for rocky mountain spotted fever (RMSF) also came back weakly positive; on further research and discussion with the infectious disease service, this was believed to be due to the test's cross-reactivity with other spirochetal infections, such as *B. burgdorferi* [7]. A repeat of the RMSF IgM was negative, as was the IgG. Based on this, along with the recent history of engorged tick bite, arthralgias, panuveitis, and nonspecific erythematous rash, the diagnosis of Lyme carditis was made, and doxycycline (100 mg twice daily) in combination with prednisone (50 mg once daily) was initiated

On the first day of treatment, the patient experienced increased erythema of her extremities with generalized pruritus, subjective fever, and fatigue consistent with a Jarisch-Herxheimer reaction. This was treated with aspirin every four hours, which resulted in complete resolution of the symptoms. Her left eye panuveitis, arthralgias, and shortness of breath all resolved with ongoing pharmacotherapy.

Although a majority of Lyme carditis cases have associated conduction abnormalities, the absence thereof should not rule out *Borrelia *species infection entirely. Lyme disease notoriously has a very broad spectrum of clinical presentations that are often nonspecific, and even ELISA in the early disease state is unreliable. Additionally, even though Lyme is more commonly associated with the northeastern United States, geography alone should not remove it from the differential - highlighted by our patient being a lifelong resident of Florida without recent travel.

## Conclusions

Lyme borreliosis is a tickborne illness caused by the spirochete *B. burgdorferi*, which is endemic to the northeast, midwest, and northwest of the United States. This systemic illness varies in presentation and severity, with a broad manifestation encompassing constitutional symptoms, erythema migrans skin lesions, heart block, myopericarditis, uveitis, arthritis, meningoencephalitis, and chronic dermatitis. Lyme disease should be suspected in those who present with overlapping symptoms, outdoor activity in endemic regions, or in association with a tick bite. Diagnosis is confirmed using two-step serologic testing with ELISA, followed by a Western blot. Lyme carditis presents as an early disseminated disease (Stage 2) with preceding erythema migrans. Though the incidence of Lyme carditis is relatively low in the United States, cardiac conduction abnormalities develop in a vast majority of those cases. The case presented highlights a rare presentation wherein an immunocompromised resident of Florida presented with an acute onset of heart failure with reduced ejection fraction, hypervolemia with hypoxia, injected conjunctiva, and global vision loss of the left eye in the setting of Lyme carditis without conduction abnormalities. She was given a one-time dose of intraocular vancomycin and ceftazidime empirically and was subsequently initiated on oral doxycycline and prednisone taper. While undergoing therapy, symptoms consistent with a Jarisch-Herxheimer reaction occurred and were treated with aspirin and diphenhydramine. The patient was discharged with near complete resolution of her presenting symptoms. This case underscores the importance of keeping a wide differential and taking a thorough history when evaluating a patient with new-onset systolic dysfunction. It further highlights the potential ambiguity in both laboratory and clinical findings for Lyme carditis, which may make formalizing the diagnosis more difficult. Overall, Lyme carditis has a wide variety of manifestations that may be further complicated by a patient's level of immunocompromise.
